# Laparoscopic uterine ventrosuspension procedure by using anterior mesh incorporated with bilateral round ligamentopexy: Kahyaoglu technique

**Published:** 2020-03-27

**Authors:** S Kahyaoglu, Y Ustun

**Affiliations:** Department of Gynecological Endoscopy, Ankara Dr. Zekai Tahir Burak Women’s Health Education and Research Hospital, University of Health Sciences, Ankara, Turkey.

**Keywords:** Laparoscopy, gynecological surgery, prolapse, urogynaecology surgical treatment, surgical technique

## Abstract

A number of vaginal and abdominal surgical techniques have been described for the treatment of apical uterine prolapse. A 38-year-old parous woman has been operated laparoscopically due to grade 3 apical uterine prolapse. A new surgical technique for the treatment of apical uterine prolapse performed by incorporation of the lateral arms of anteriorly anchored uterine polypropylene mesh to bilateral round ligaments has been developed. Due to the young age of the patient and lack of any other uterine pathology, a laparoscopic uterine ventrosuspension procedure combined with bilateral round ligamentopexy has been performed as a low risk uterine prolapse surgery.

## Introduction

Pelvic organ prolapse is the clinical result of repetitive traumatic insults to pelvic support components based on a genetic susceptibility to vulnerable connective tissues (Maher et al., 2016). Several surgical restorative techniques have been defined for correction of pelvic organ prolapse regarding the defective site of pelvic support elements. Uterine descent is typically defined as an apical defect of pelvic support which has been demonstrated by De Lancey as level 1 defect. Vaginal hysterectomy with vaginal sacrospinous ligamentopexy procedure is performed when uterine prolapse occurs at an older age, with completed fertility and desire for hysterectomy. In young women, apical uterine prolapse can be treated via vaginal or abdominal routes by performing vaginal sacrospinous hysteropexy or abdominal sacrohysteropexy, respectively. Laparoscopic lateral suspension and laparoscopic high uterosacral ligament suspension are two other surgical procedures which can be utilized by surgeons with satisfactory results for patients suffering from apical uterine prolapse (Hsieh, 2011; Veit-Rubin et al., 2016; Jan and Ghai, 2018; Jan and Ghai, 2019). In this video presentation, we describe a new surgical technique performed for the correction of apical prolapse in which we incorporated the lateral arms of the anteriorly anchored uterine mesh to bilateral round ligaments combined with bilateral round ligamentopexy.

## Methods

**Figure 1:**
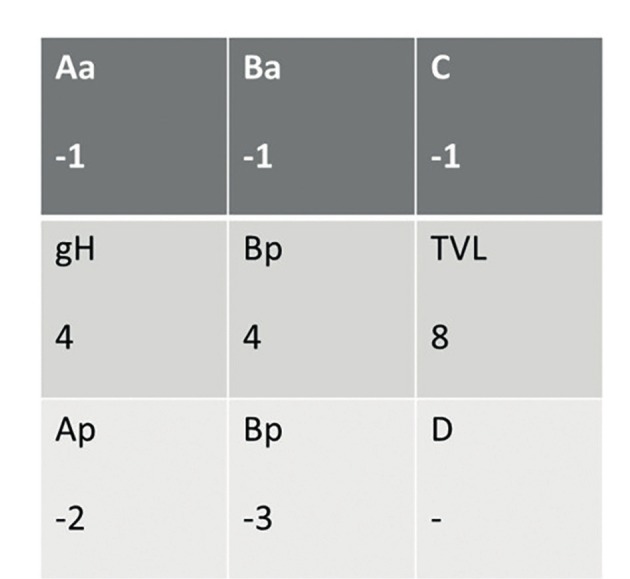
­Preoperative pelvic organ prolapse quantification (POP-Q) grades of the patient.

**Figure 2:**
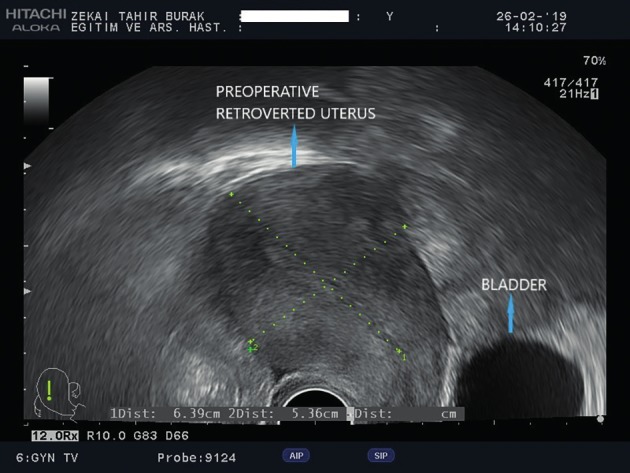
Preoperative transvaginal ultrasonographic view of retroverted uterus.

**Figure 3:**
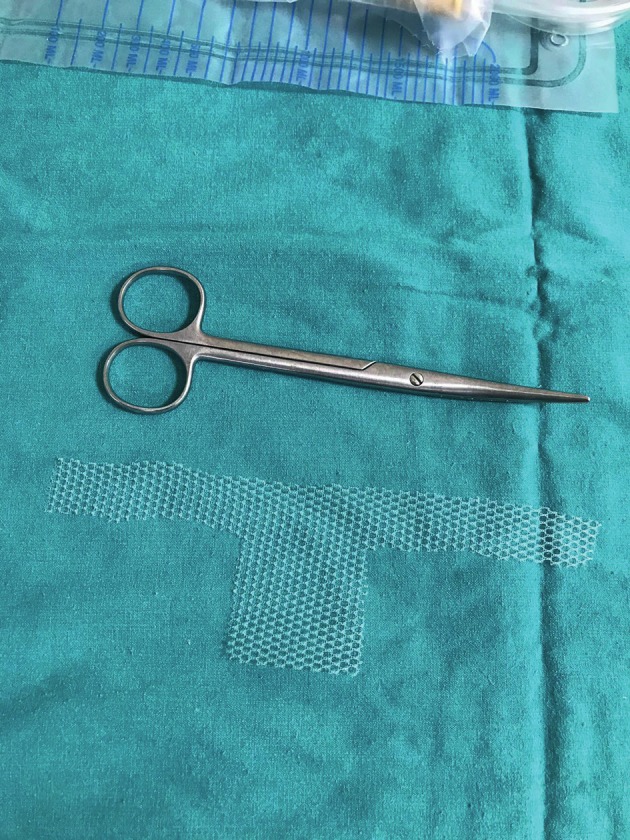
Photograph of the 4*5 cm diamond shaped nonabsorbable mesh (Parietene TM Macroporous Mesh, Monofilament Polypropylene, Covidien TM ) with lateral arms of 2 cm in longevity .

A written informed consent was obtained from the patient before the procedure. A 38-year-old gravida 4, para 4 woman attended our gynecology unit with a prolapsed uterus. One year earlier, she had undergone anterior colporrhaphy and transobturator tape insertion procedures due to cystocele and urinary incontinence and her past medical history was otherwise uneventful. All four births had occurred vaginally and this was the only risk factor for uterine prolapse in her history. Upon vaginal examination; grade 3 apical uterine prolapse, grade 1 cystocele and grade 1 rectocele were determined. According to the pelvic organ prolapse-quantification (POP-Q) classification, the levels of uterovaginal landmarks were detected as follows: Aa:-1 Ba:-1, Ap:-2, Bp:-3, C:+1, TVL:8. Bilaterally normal appearing ovaries and a retroverted uterus including a 2 cm intramural adenomyoma anteriorly were seen upon transvaginal ultrasonography (Figure 1). She had a slightly increased protrombin time level of 15.9 seconds and INR value of 1.38 related to partially decreased level of factor XII. Due to the young age of the patient and lack of any other uterine pathology, we decided to perform a laparoscopic uterine ventrosuspension procedure combined with bilateral round ligamentopexy as a new and low risk uterine prolapse surgery method. Six units of fresh frozen plasma packs have been intravenously administered before and during surgery due to the slightly increased INR level. During laparoscopic surgery, an assistant introduced a V-Care uterine manipulator (Conmed, Utica, New York, USA) to the cervix and pushed the uterus cranially and dorsally to create a sufficient retroversion for suturing. The peritoneal reflections just below both round ligaments were cut and dissected and a bladder flap was created. A 4x5 cm diamond shaped nonabsorbable mesh (ParieteneTM Macroporous Mesh, Monofilament Polypropylene, CovidienTM) was anchored to the anterior part of the cervix and upper vagina by using a 2/0 prolene nonabsorbable suture (Figure 2). The lateral arms of the centrally anchored mesh which were cut 2 cm in length were incorporated to the round ligaments by using the same suture material. Suturing was performed in a continuous manner starting from the lateral edge of the round ligaments and progressing along the round ligaments until incorporation of lateral arms of the mesh to the round ligaments at the level of the junction between the round ligaments and uterus, before returning to the starting point. At this step, the suture edges were pulled to achieve an effective shortening of the round ligaments accompanied by pulling the anteriorly anchored mesh. The peritoneum was then closed continuously by using a 2/0 polyglactin suture, ensuring complete coverage of the mesh. A bilateral tubal ligation procedure was also performed at the patient’s request. (Video l: qrco.de/kahyaoglu). Total operation time was one and a half hours and estimated blood loss was ~100 ml. No intraoperative or postoperative complications were encountered. Postoperatively, the levels of uterovaginal landmarks were detected as follows: Aa:-2 Ba:-3, Ap:-2, Bp:-3, C:-5, TVL:8 based on the POP-Q classification. The traction effect of anterior polypropylene mesh incorporated to shortened round ligaments resulted in improved Ba and Bp levels following the surgery. A satisfying uterine suspension was obtained following this new and intrinsically low risk surgical method. Correction of retroversion and echogenicity of anteriorly anchored mesh were demonstrated by transvaginal ultrasonography on postoperative day 1 (Figure 3). The postoperative follow-up of the patient was uneventful and she was discharged on the day after surgery. She was again seen for a follow-up 3 months after the surgery and sustained postoperative improved POP-Q levels without any mesh complications were confirmed upon gynecological examination, demonstrating short term efficacy and safety of the surgical procedure.

**Figure 4:**
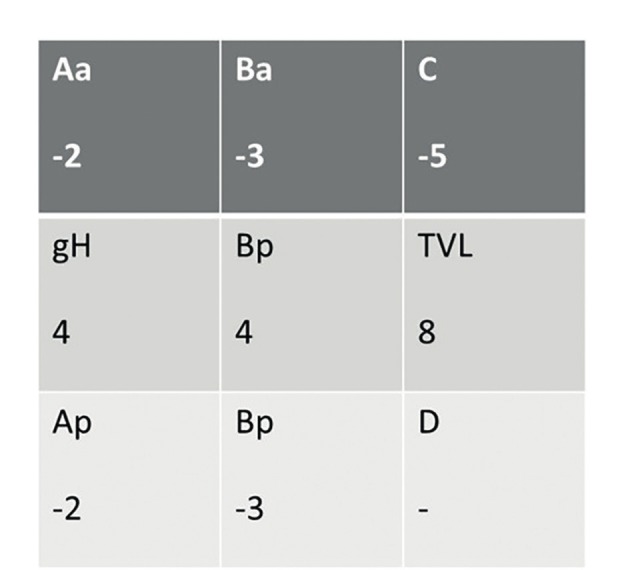
Postperative pelvic organ prolapse quantification (POP-Q) grades of the patient.

**Figure 5:**
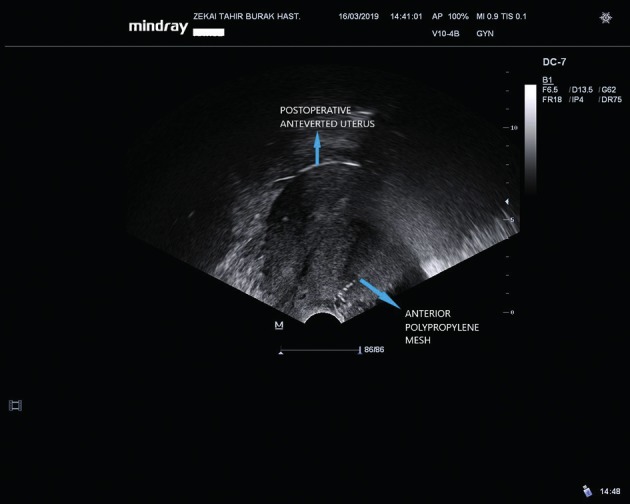
Postoperative transvaginal ultrasonographic view of anteverted uterus and anteriorly anchored polypropylene mesh echogenicity.

## Discussion

Pelvic organ prolapse (POP) is a highly prevalent condition among women due to various factors such as vaginal deliveries, genetics, obesity and other conditions which cause increased intraabdominal pressure. Uterine preserving surgeries have recently gained popularity for the treatment of POP due expectation of maintenance of sexuality, reluctance for loss of reproductive organs and the desire to preserve fertility. Besides, surgical treatment modalities which are performed for apical pelvic organ prolapse should generally be performed after completion of childbearing due to the lack of reassuring high quality studies demonstrating the safety of pregnancy following surgical treatment. A few successful pregnancies following abdominal sacrohysteropexy have previously been reported but long term durability of surgery is unknown (Albowitz et al., 2014; Balsak et al., 2015). Patients who get pregnant after surgery for apical uterine prolapse may be prone to pelvic pain or mesh complications during pregnancy and childbirth. Future studies are needed to determine pregnancy outcomes following apical prolapse surgery. Open sacrocolpopexy/ sacrohysteropexy has been considered the gold standard in the treatment of apical POP which preferentially ought to be performed by minimally invasive surgery. Although vaginal sacrospinous fixation and abdominal sacrohysteropexy have been utilized with satisfying success rates for relieving apical uterine prolapse; pelvic pain and increased risk for injury of retroperitoneal vessels, nerves, and neighboring tissues are potential complications of these sophisticated surgical techniques. In a retrospective study, surgical outcomes of 245 POP patients who have been treated with uterine- preserving laparoscopic lateral suspension with mesh have been evaluated for a median follow-up of 7.5 years. The authors have concluded that lateral suspension is a safe technique with promising results and low complication rates. They also stated that it may be an alternative to sacral hysteropexy for high- risk patients (Veit-Rubin et al., 2016). Laparoscopic lateral suspension of the apically prolapsed uterus, which has been defined before, is a good alternative to sacrohysteropexy and sacrocolpopexy. However, during a laparoscopic lateral suspension (LLS) procedure, the surgeon should move the laparoscopic grasping instrument from abdominal port towards the fixation site which is underneath the round ligament and this location is in relatively close proximity to external iliac vessels. Secondly, unlike the new technique that we have described, longer mesh arms are used for lateral suspension surgery. Utilization of a smaller mesh area may be an advantage of our new surgical technique. LLS with or without hysterectomy is a safe technique with high patient satisfaction. In a retrospective study, surgical outcomes of 339 women with symptomatic anterior and/or apical pelvic organ prolapse (POP) and an intact uterus have been evaluated to compare differences between LLS with supracervical hysterectomy (LLSHE) and uterine preservation (LLSUP). The authors have concluded that the uterus-preserving approach appears to result in better anatomic outcome for the anterior compartment, better subjective outcome, and higher patient satisfaction (Veit-Rubin et al., 2019). In a systematic review, surgical outcomes of uterine-preserving surgeries performed for the repair of pelvic organ prolapse have been compared. Based on 99 mostly heterogenous studies and scarce data directly comparing different hysteropexy types; different types of hysteropexies have a wide range of POP recurrence rates and adverse effects (Meriwether et al., 2019).

In this article, we describe a new surgical technique for the treatment of apical uterine prolapse which was performed by incorporation of the lateral arms of anteriorly anchored uterine polypropylene mesh to bilateral round ligaments. Unlike laparoscopic sacrohysteropexy, pectopexy and lateral fixation procedures; the surgical steps performed during this new surgical technique do not increase the risk of damage to neighbouring vessels, nerves or bones. Although laparoscopic sacrohysteropexy is an effective procedure for alleviating apical uterine prolapse due to achievement of a strong pelvic support, low risk of recurrence and fertility preservation; it requires high level skills in laparoscopic surgery which precludes its utilization by a wide range of gynecologists (Hengrasmee and Lam, 2015). During sacrohysteropexy surgery, dissection at the level of the promontory may be challenging, particularly in obese women, and anatomical variation of abdominal vessels poses increased risk for surgical complications like serious neurological or ureteral morbidity and life-threatening vascular injuries even for experienced laparoscopic surgeons. However, it should be appreciated that our new technique still requires laparoscopic expertise and careful needle control and tissue dissection to avoid neighboring vascular injury, to appropriately transfix the mesh and to appropriately bury it. Utilization of a very small amount of surgical mesh during this surgery to achieve the necessary and efficient traction force to an apically prolapsed uterus seems to be an surgical advantage regarding mesh complications. In the future, as a modification of this surgical technique, partial dissection of the peritoneum near the uterine entry site may be enough to anchor the lateral arms of the anteriorly placed mesh to the round ligaments. In addition, a learning curve for this new technique might be shorter than previously described vaginal sacrohysteropexy and abdominal sacrocolpopexy procedures. Although not identical, this new uterine suspension technique resembles the mini-sling operation which is performed for alleviating stress incontinence; they both use a small amount of nonabsorbable mesh material. Before contemplating performing this new surgical technique for apical uterine prolapsus treatment, future studies comparing the efficacy, side effects, patient preferences and long term durability of this new surgical technique with previously described ones are needed.

## Conclusion

Abdominal and vaginal procedures have previously been developed for the treatment of apical uterine prolapse. Each surgical procedure includes its own surgical risks and short/long term durability. Laparoscopic uterine ventrosuspension by using anterior polypropylene mesh incorporated with bilateral round ligamentopexy can easily be utilized by gynecologists without exposing patients to unnecassary surgical risks such as vascular and neurological injuries. However; long term efficacy, safety and durability of this new surgical technique should be confirmed by further studies before it is performed routinely for apical uterine prolapsus.

**Video scan (read QR):**
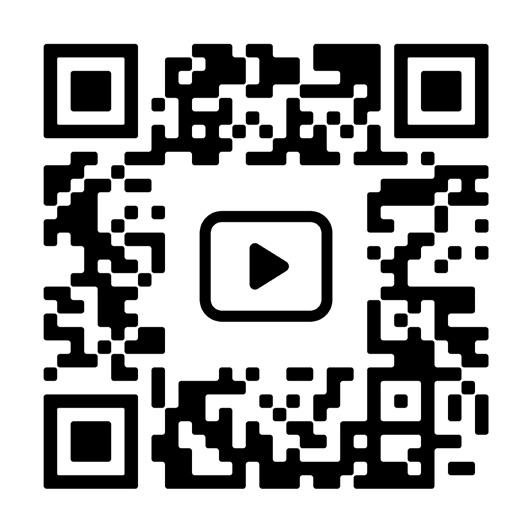


Supplementary video-link: qrco.de/kahyaoglu
